# Bodily maps of uncertainty and surprise in musical chord progression and the underlying emotional response

**DOI:** 10.1016/j.isci.2024.109498

**Published:** 2024-04-04

**Authors:** Tatsuya Daikoku, Masaki Tanaka, Shigeto Yamawaki

**Affiliations:** 1 Graduate School of Information Science and Technology, The University of Tokyo, Tokyo, Japan; 2 Centre for Neuroscience in Education, University of Cambridge, Cambridge, UK; 3 Center for Brain, Mind and KANSEI Sciences Research, Hiroshima University, Hiroshima, Japan

**Keywords:** Biological sciences, Neuroscience, Sensory neuroscience

## Abstract

Music has profoundly shaped the human experience across cultures and generations, yet its impact on our minds and bodies remains elusive. This study examined how the perception of musical chord elicits bodily sensations and emotions through the brain’s predictive processing. By deploying body-mapping tests and emotional evaluations on 527 participants exposed to chord progressions, we unveiled the intricate interplay between musical uncertainty, prediction error in eliciting specific bodily sensations and emotions. Our results demonstrated that certain chord progressions elicit cardiac and abdominal sensations, linked to interoception, and associated with aesthetic appreciation and positive valence. These findings highlight the crucial role of musical uncertainty and prediction error in emotional response and sound embodiment. This study offers insight into the potential connection between music-induced interoception and mental well-being, underscoring the musical effects on our minds and bodies.

## Highlights

• This study examined the body map of musical chord progressions
• Musical uncertainty and surprise shape emotional response and body map
• Temporal fluctuations in prediction affect heart and abdominal sensations
• Heart sensation positively correlates with valence in a specific chord progression

## Graphical abstract

**Figure:**
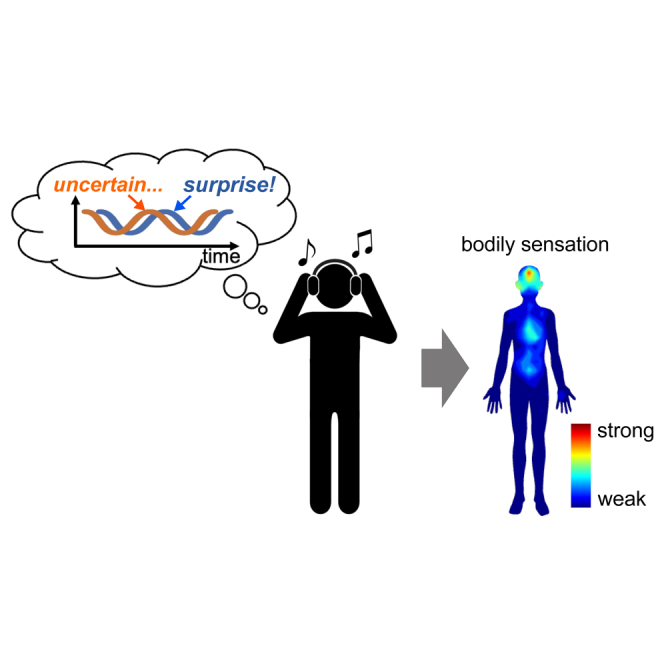

## Introduction

Music, an omnipresent force throughout human history, has deeply influenced our minds and bodies.^1^ Its pervasive and far-reaching effects have captivated not only the scientific community but also a broad audience, spanning diverse cultures and generations. Despite centuries of inquiry into the myriad ways in which music influences our minds and bodies, this intriguing question remains largely unanswered and offers fertile ground for new discoveries and insights.

A promising avenue to unlock this enigma revolves around the unique bodily sensations elicited by music.^2^^,^^3^ Beyond the external sensory perceptions (i.e., exteroception) like an auditory system music elicits internal bodily perceptions such as interoception (e.g., heartbeat acceleration or the spine-chilling thrill) and proprioception (e.g., the constrictive sensation in one’s chest when hearing sorrowful music).^4^^,^^5^^,^^6^ Particularly, interoceptive awareness, driven by shifts in interoceptive sensations, has been identified as being integral to our mental^7^ and potentially associated with music emotion and its embodiment.^8^^,^^9^ Although research has richly explored how music hearing through exteroception influences our emotions, the intricate interplay between music and internal bodily perceptions involved in emotions remains largely unexplored.

The emotions are tightly interwoven with our bodily sensation. A past study revealed that different emotions can be discerned through the mapping of emotion-triggered bodily sensations.^10^ They suggested that diverse emotions have distinct body topographies with negative emotions such as fear, anger, sadness, and anxiety activating the upper side of the body and positive ones such as happiness and love casting a wider range of activation. This implies that emotions are embodied, manifesting spatially within the body. Further, a past study revealed interoceptive accuracy was associated with emotion-triggered bodily sensations.^11^ They suggest that the awareness of one’s internal bodily states might play a crucial role as a required messenger of sensory information during the affective process.

The relationship between bodily sensations and emotions can be elucidated from the perspective of the brain’s predictive processing. Predictive processing operates on the principle that our brain constantly anticipates and predicts sensory inputs based on prior experiences.^12^ When there’s a mismatch between the predicted and actual sensory input, a prediction error is generated. Interoception, which refers to the brain’s perception of internal bodily states, plays a pivotal role in this context. The brain generates emotions by minimizing prediction errors between the anticipatory signals derived from its internal model and the sensory signals through exteroceptive and interoceptive sensations.^13^ Within the framework of music, when our musical predictions are not met, it can lead to a visceral, interoceptive response.^6^ For instance, if we anticipate a musical chord progression based on our prior experiences and the music deviates from this expectation, it can generate a prediction error.^14^^,^^15^ This error might manifest as a sudden change in heartbeat or a rush of emotions associated with surprise, both of which are interoceptive responses. The consequent interoceptive prediction error may be resolved by updating interoceptive priors, possibly through deep breathing or other measures. Evidence has revealed that interoceptive awareness arises from the updating of prediction errors.^16^^,^^17^^,^^18^ Uncertainty, in this framework, pertains to the degree of unpredictability associated with a sensory input. Low uncertainty can amplify the brain’s reliance on its predictions, leading to stronger surprise-related interoceptive reactions when those predictions are not met.

Regarding music prediction and the elicited emotion, recent research has illuminated the critical interplay of both predictive uncertainty and surprise (prediction error).^14^^,^^19^^,^^20^^,^^21^ In a study by Cheung et al.,^14^ they developed a predictive model that mathematically decodes the relationship between uncertainty and surprise in music. Analyzing 80,000 chords from US Billboard pop songs, they anchored their study in the framework of Western tonal harmony, viewing it as a representation of musical syntax. The study found that chords combining low uncertainty with high surprise or those combining high uncertainty with low surprise were the most pleasurable. Neurologically, this effect correlated with activity in regions such as the amygdala, hippocampus, and auditory cortex. Notably, dopaminergic pathways in the nucleus accumbens, which responded positively to uncertainty, seemed to play a pivotal role. Their findings echoed Berlyne’s seminal model, where pleasure forms an inverted-U relationship with factors like complexity. However, the multifaceted nature of musical pleasure, as Cheung et al. illustrated, suggests that our musical experiences are shaped by a web of interacting variables such as uncertainty-weighted inverted U relationship, rather than isolated factors. In recent decades, the relationship between exteroceptive audition (auditory cortex), and music emotion based on predictive processing is progressively being elucidated.^22^^,^^23^^,^^24^^,^^25^^,^^26^ However, the associations of music emotion with interoception and bodily sensations represented as the body maps continue to be an under-researched frontier.

This study embarks on this unexplored voyage. We aspire to elucidate how perceptions of musical chord progressions are embodied, giving rise to emotional experience, grounded in the framework of the bodily mapof emotions. Specifically, we aim to determine what types of musical chords, through the lens of predictive processing, evoke interoceptive bodily sensations, particularly those related to the cardiac and abdominal (stomach) regions. The cardiac region, being the seat of our heartbeat, is often associated with visceral and emotional reactions to stimuli such as surprise,^27^ while the abdominal region, encompassing the stomach, might be linked to gut feelings^28^ or intuitive affective reactions such as disgust.^29^ Further, evidence has shown that these two regions are specifically known to be associated with interoceptive awareness and interoceptive prediction error.^13^^,^^16^^,^^30^ Recent evidence has suggested that interoceptive experience may largely reflect limbic predictions about the expected state of the body that are constrained by visceral sensations.^31^

Given the intricate relationship between music prediction, emotions, and cardiac/abdominal sensations, we hypothesize that different musical predictions might elicit distinct patterns of sensations in these two regions, potentially offering insights into the nuanced ways in which music interacts with our internal bodily perceptions. Rooted in predictive processing principles, our central hypothesis is that two factors: uncertainty-weighted prediction error and its fluctuations (temporal dynamics) critically contribute to interoception evoking bodily sensations in the cardiac and abdominal regions.

With respect to the element of uncertainty-weighted prediction error, based on the uncertainty-weighted inverted U relationship characterizing musical pleasure, we posited that chords combining low uncertainty with high surprise or those combining high uncertainty with low surprise induce the interoception evoking bodily sensations. Notably, it has been known that the increasing complexity (uncertainty) of regularities requires an increasing amount of knowledge about musical regularities to make precise predictions about upcoming musical events^32^^,^^33^^,^^34^ and that the correct and precise predictions may be perceived as more rewarding.^35^ Consequently, for the general people who are not experts in music, chords combining low uncertainty with high surprise might be particularly salient in accurately recognizing prediction errors, thereby potentially amplifying interoceptive bodily sensations and the associated emotions.^36^

Concerning the element of the fluctuations (temporal dynamics) of uncertainty and surprise, the facilitation of interoceptive bodily sensations by uncertainty-weighted prediction error could be modulated by preceding musical contexts. That is, while it has been known that musical pleasure forms a two-dimensional inverted U curve based on uncertainty and surprise, music entails extended contexts that cannot be fully captured by the simple prediction and uncertainty between one chord and the next. The music context can influence the perception of even the same two-chord sequence, resulting in different emotional experiences depending on the surrounding context. Consequently, in addition to the two dimensions of uncertainty and surprise, it is necessary to elucidate musical prediction and emotion through a three-dimensional model that takes into account the preceding contexts (more dynamics). These temporal dynamics of uncertainty-weighted prediction error could offer a more nuanced understanding of the emotions and bodily sensations intrinsic to music as a temporal art form.

## Results

All participants (*N* = 527) completed body-mapping tests and the following emotional judgements in every one of the eight types of 4-chord progression (see the STAR Methods section for details). These chord progressions were generated using a statistical-learning model^37^^,^^38^ to compute the Shannon information content and entropy, based on transitional probabilities^39^ of each chord using a corpus of 890 pop songs from the US Billboard.^40^ Entropy gauges the perceptual uncertainty a listener feels in predicting an “upcoming” chord based on prior chords, while information content quantifies the surprise experienced upon hearing the actual chord.

**Figure 1:**
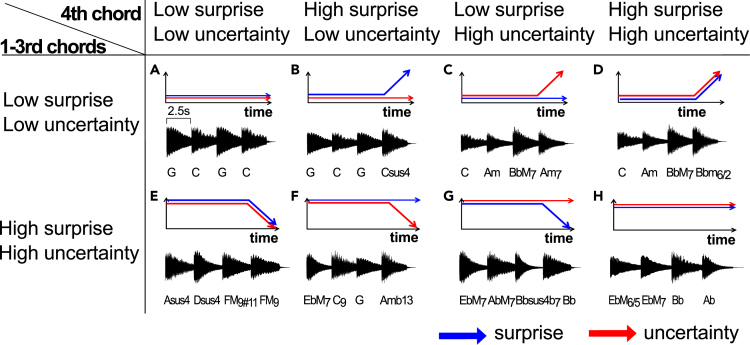
Examples of chord progressions used in the experiment, with controlled surprise and uncertainty The eight types of chord progressions were generated using a statistical learning model^38^ to compute the Shannon information content and entropy, based on transitional probabilities of each chord using a corpus of 890 pop songs from the US Billboard.^40^ Entropy gauges the perceptual uncertainty a listener feels in predicting an “upcoming” chord based on prior chords, while information content quantifies the surprise experienced upon hearing the actual chord. Each type is characterized by varying degrees of uncertainty (red lines) and surprise (blue lines). That is, they are (1) sLuL-sLuL sequence representing the condition where the 1st–3rd chords have low surprise and uncertainty and the 4th chord has low surprise and uncertainty, (2) sLuL-sHuL sequence representing the condition where the 1st–3rd chords have low surprise and uncertainty and the 4th chord has high surprise and low uncertainty, (3) sLuL-sLuH sequence representing the condition where the 1st-3rd chords have low surprise and uncertainty and the 4th chord has low surprise and high uncertainty, (4) sLuL-sHuH sequence representing the condition where the 1st–3rd chords have low surprise and uncertainty and the 4th chord has high surprise and uncertainty, (5) sHuH-sLuL sequence representing the condition where the 1st–3rd chords have high surprise and uncertainty and the 4th chord has low surprise and uncertainty, (6) sHuH-sHuL sequence representing the condition where the 1st-3rd chords have high surprise and uncertainty and the 4th chord has high surprise and low uncertainty, (7) sHuH-sLuH sequence representing the condition where the 1st-3rd chords have high surprise and uncertainty and the 4th chord has low surprise and high uncertainty, and (8) sHuH-sHuH sequence representing the condition where the 1st–3rd chords have high surprise and uncertainty and the 4th chord has high surprise and uncertainty. In the end, four of the 8 types began with three chords, each with low surprise and uncertainty (sLuL: A–D), while the other four began with three chords each displaying high surprise and uncertainty (sHuH: E–H). For each chord progression, the last (i.e., 4th) chord was generated with a 2x2 pattern, varying in uncertainty and surprise. The first pattern exhibited both low surprise and low uncertainty (sLuL: A and E), the second had low uncertainty but high surprise (sHuL: B and F), the third showcased low surprise with high uncertainty (sLuH: C and G), and the fourth possessed both high surprise and uncertainty (sHuH: D and H).

Using this model, we generated the 92 unique chord progressions encompassed within the eight types of chord progressions. Each type is characterized by varying degrees of uncertainty (red lines in Figure 1) and surprise (blue lines in Figure 1). That is, they are (1) sLuL-sLuL sequence (Figure 1A) representing the condition where the 1st–3rd chords have low surprise and uncertainty and the 4th chord has low surprise and uncertainty, (2) sLuL-sHuL sequence (Figure 1B) representing the condition where the 1st-3rd chords have low surprise and uncertainty and the 4th chord has high surprise and low uncertainty, (3) sLuL-sLuH sequence (Figure 1C) representing the condition where the 1st-3rd chords have low surprise and uncertainty and the 4th chord has low surprise and high uncertainty, (4) sLuL-sHuH sequence (Figure 1D) representing the condition where the 1st–3rd chords have low surprise and uncertainty and the 4th chord has high surprise and uncertainty, (5) sHuH-sLuL sequence (Figure 1E) representing the condition where the 1st-3rd chords have high surprise and uncertainty and the 4th chord has low surprise and uncertainty, (6) sHuH-sHuL sequence (Figure 1F) representing the condition where the 1st-3rd chords have high surprise and uncertainty and the 4th chord has high surprise and low uncertainty, (7) sHuH-sLuH sequence (Figure 1G) representing the condition where the 1st-3rd chords have high surprise and uncertainty and the 4th chord has low surprise and high uncertainty, and (8) sHuH-sHuH sequence (Figure 1H) representing the condition where the 1st-3rd chords have high surprise and uncertainty and the 4th chord has high surprise and uncertainty.

In the end, four of the 8 types began with three chords, each with low surprise and uncertainty (sLuL: A–D in Figure 1), while the other four began with three chords each displaying high surprise and uncertainty (sHuH: E–H in Figure 1). For each chord progression, the last (i.e., 4th) chord was generated with a 2x2 pattern, varying in uncertainty and surprise. The first pattern exhibited both low surprise and low uncertainty (sLuL: Figures 1A and 1E), the second had low uncertainty but high surprise (sHuL: Figures 1B and 1F), the third showcased low surprise with high uncertainty (sLuH: Figures 1C and 1G), and the fourth possessed both high surprise and uncertainty (sHuH: Figures 1D and 1H). The thresholds for the high and low values were established based on the top and bottom 20% of all data points for each uncertainty and surprise. Multiple chord progressions were generated for each of the eight types, and the chord progression employed was randomly selected for each participant.

Participants were exposed to these eight types of chord progressions in random order. Following each listening session, they were asked to respond within 10 s with clicks to the position in the body where they felt from the chords, using the body image presented on the screen. Two types of emotional judgements were administered. The first comprised multiple-choice categorical judgements; that is, in each type of chord progression, participants were required to select the best 5 emotional categories in ranking elicited by each sound from a list of 33 categories. The second kind comprised nine-point Likert scales of valence and arousal. We compared the topography of chords and the corresponding emotional responses among the eight types of chord progressions.

### Body map

**Figure 2:**
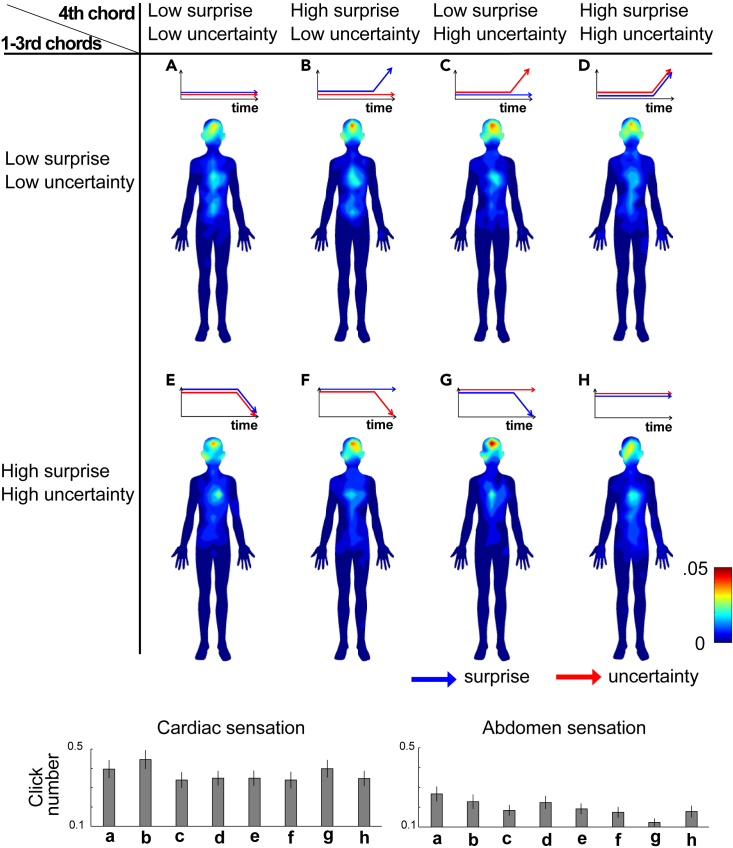
Bodily map of musical chord progressions and the click numbers of cardiac and abdomen or each type of chord progression The blue-to-red gradients represent the number of clicks. The blue and red arrows represent the surprise and uncertainty values, respectively. Four of the 8 types began with three chords, each with low surprise and uncertainty (A–D), while the other four began with three chords each displaying high surprise and uncertainty (E–H). For each chord progression, the last (i.e., 4th) chord was generated with a 2x2 pattern, varying in uncertainty and surprise. The first pattern exhibited both low surprise and low uncertainty (A and E), the second had low uncertainty but high surprise (B and F), the third showcased low surprise with high uncertainty (C and G), and the fourth possessed both high surprise and uncertainty (D and H). The below bar figures represented the numbers of clicks at cardiac and abdomen areas during listening to each type of musical chord progression. Data are represented as mean ± SEM.

Grand averages of the bodily mapand the clicked positions were shown in Figures 2 and S2 in the supplemental information, respectively. All the anonymized raw data files and all the results of statistical analyses including the descriptives have been deposited to an external source (https://osf.io/cyqhd/). As illustrated in Figure 2, each chord progression revealed a distinct body map. The contrast analyses suggested that the sLuL-sLuL sequence (Figure 2A), representing the condition where the 1st-3rd chords have low surprise and uncertainty and the 4th chord has low surprise and uncertainty, provoked pronounced bodily sensations localized to the abdomen (T = 2.42, p = 0.016, Cohen’s d = 0.11, 95% CI = 0.0078–0.0735). Further, the sLuL-sHuL sequence (Figure 2B), representing the condition where the 1st-3rd chords have low surprise and uncertainty and the 4th chord has high surprise and low uncertainty, provoked pronounced bodily sensations localized to the heart, as compared with other types of chord sequences (T = 2.04, p = 0.042, Cohen’s d = 0.09, 95% CI = 0.0016–0.0843).

The click data may reveal dependencies between sensations in specific areas in terms of spatial autocorrelation, suggesting individuals might indicate sensations across extensive areas. Additionally, there may be individual variations, where the overall intensity or extent of sensation reported can differ among participants. To investigate these variances and dependencies, we employed a generalized linear mixed model analysis (GLMM).^41^ Specifically, we examined the relationships between total clicks and those in the cardiac and abdominal areas, as well as the interplay between cardiac and abdominal clicks. A threshold of p < 0.05 was set for statistical significance. For instance, if the number of abdominal clicks changes regardless of the cardiac clicks, this unpredictability (statistically non-significant) suggests that sensations in these two areas are independent. Conversely, when it comes to the relationship between clicks in specific regions and total clicks, if one mostly clicks on the heart region, the overall click count should be predictable (statistically significant) by the number of cardiac clicks. This would imply that an increased number of heart clicks isn’t merely a result of clicking on various areas. However, if the total click count varies regardless of heart clicks, then a correlation is unlikely.

Our results indicate that cardiac sensations are not influenced by abdominal sensations (z = 0.726, p = 0.468, 95% CI = −0.033 to 0.015), with a relatively low individual variability (variance = 0.119, SD = 0.019) (refer to Table S2; Figure S6). As for the relationship between cardiac sensations and overall bodily sensations, cardiac sensations significantly influence with overall sensations (z = 25.750, p < 0.001, 95% CI = 0.140 to 0.163), exhibiting limited individual variability (variance = 0.271, SD = 0.031) (see Table S3; Figure S7). In the case of abdominal sensations versus overall sensations, there is a significant dependency (z = 18.963, p < 0.001, 95% CI = 0.074 to 0.092) with limited individual variability (variance = 0.089, SD = 0.015) (refer to Table S4; Figure S8). For head sensations in relation to overall sensations, there exists a significant dependence (z = 38.124, p < 0.001, 95% CI = 0.283 to 0.313), though with more noticeable individual differences (variance = 1.048, SD = 0.087) (see Table S5; Figure S9).

The Shapiro–Wilk test for normality showed the violations of the assumption of normality on all the data (p < 0.001). Hence, we applied the Friedman’s non-parametric Repeated-measure analysis of variance (ANOVA) for within-subject factor “types of chord progressions”. The clicks at the abdomen position showed significant main effects (χ^2^ = 16.7, df = 7, p = 0.019). The Durbin-Conover post hoc test detected that the abdomen sensation was stronger in the sLuL-sLuL sequence (Figure 2A) than in the sHuH-sLuH sequence (Figure 2G) representing the condition where the 1st–3rd chords have high surprise and uncertainty and the 4th chord has low surprise and high uncertainty (p < 0.001). The clicks at the head position showed significant main effects (χ^2^ = 25, df = 7, p < 0.001). The Durbin-Conover post hoc test detected that the head sensation was stronger in the sLuL-sLuH sequence (Figure 2C) than in the sHuH-sHuH sequence (p < 0.001, Figure 2A), the sLuL-sHuL sequence (p < 0.001, Figure 2B), and the sHuH-sLuL sequence (p = 0.005, Figure 2E). Further, the head sensation was stronger in the sHuH-sLuH sequence (Figure 2G) than in the sLuL-sLuL sequence (p < 0.001, Figure 2A), the sLuL-sHuL sequence (p = 0.005, Figure 2B), and the sHuH-sLuL sequence (p = 0.003, Figure 2E).

### Emotion in response to musical chord progressions

All the results of statistical analyses and the descriptives of the multiple-choice categorical judgements and nine-point Likert scale of valence and arousal have been deposited to an external source (https://osf.io/cyqhd/). The figures of the grand average data are shown in the Figures S3 and S4 in the supplemental information. The Shapiro–Wilk test for normality showed the violations of the assumption of normality on valence, arousal, and categorical emotional scores including aesthetic appreciations (p < 0.001). Hence, we applied the Friedman’s non-parametric repeated-measure ANOVA for within-subject factor: 8 types of chord progressions.

**Figure 3:**
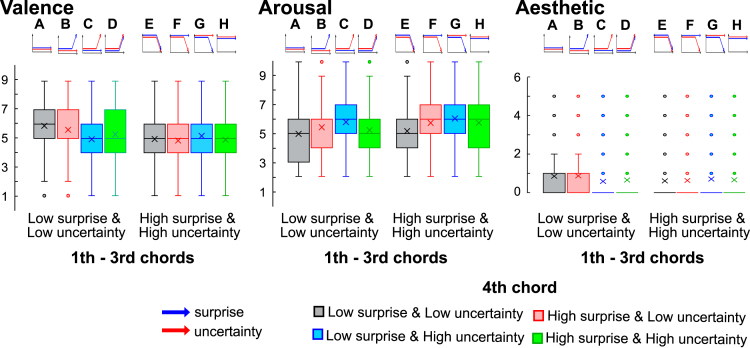
Emotional responses for musical chord progressions The blue and red arrows represent the surprise and uncertainty values, respectively. The “×” symbol in the center denotes the mean value. The horizontal line at the upper end represents the maximum value, while the horizontal line at the lower end indicates the minimum value. The bottom edge of the box marks the first quartile, the line drawn inside the box signifies the second quartile (or median), and the top edge of the box represents the third quartile. The participants rated the valence and arousal on nine-point Likert scales (1–9 points), while the aesthetic was assessed using multiple-choice categorical judgments in which participants were asked to rank and select the top 5 emotional categories. The categories ranked fifth through first were scored as 1, 2, 3, 4, and 5 point, respectively.

The ANOVA for valence showed the main effects (χ^2^ = 215, p < 0.001). The Durbin-Conover post hoc test detected that the valence was significantly more positive in the sLuL-sLuL sequence (Figure 3A, Valence) and the sLuL-sHuL sequence (Figure 3B, Valence) compared to the other types of chord progressions (Figures 3C–3H, Valence) (p < 0.001). The valence was significantly more positive in the sLuL-sLuL sequence (Figure 3A, Valence) than the sLuL-sHuL sequence (Figure 3B, Valence) (p = 0.02).

The ANOVA for arousals showed the main effects (χ^2^ = 192, p < 0.001). The Durbin-Conover post hoc test detected that the arousal was significantly lower in the sLuL-sLuL sequence (Figure 3A, Arousal) and the sLuL-sHuL sequence (Figure 3B, Arousal) than the other types of chord progressions (p < 0.01).

In categorical emotional scores, this study mainly focused on aesthetic appreciation. The ANOVA for aesthetic rating showed the main effects (χ^2^ = 27.2, p < 0.001). The Durbin-Conover post hoc test detected that the aesthetic rating was significantly higher in the sLuL-sLuL sequence (Figure 3A, Aesthetic) and the sLuL-sHuL sequence (Figure 3B, Aesthetic) than the other chord progressions (p < 0.01).

We further analyzed 32 categorical emotion scores. These results are depicted in Figure S4 of the supplemental information. Comprehensive statistical outcomes can be found at an external source: https://osf.io/cyqhd/. Our findings indicate that certain chord progressions evoke distinct emotions. Specifically, the predictable sLuL-sLuL chord progressions notably elicited emotions contrasting with excitement, including feelings of calmness, relief, satisfaction, nostalgia, and empathy (refer to Figure S4), especially when compared to other chord sequences that encompass elements of surprise or uncertainty (p < 0.01). In addition, for both the sLuL-sLuL and sLuL-sHuL sequences, which evoked aesthetic appreciation, the negative emotions of awkwardness and anxiety was notably diminished (p < 0.01).

These findings suggest that contextual temporal dynamics can lead to variations in interoceptive sensations and emotions. That is, if we only looked at first-order transitional probability and uncertainty, the sLuL-sLuL sequence (Figure 1A) and sHuH-sLuL sequence (Figure 1E) were considered the same type of chord. However, as evident from the results on emotions, the sLuL-sLuL sequence significantly exhibits higher positive valence and aesthetic appreciation compared to the sHuH-sLuL sequence.

### Relationships between bodily sensation and the emotions

**Figure 4:**
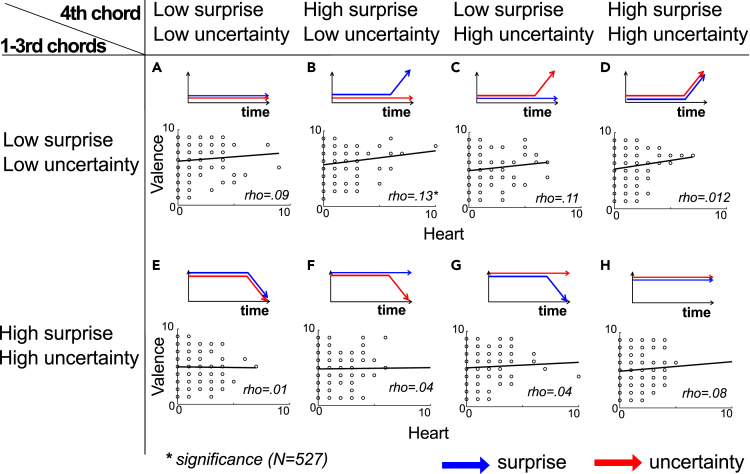
Correlation of valence rating with bodily sensations at cardiac and abdomen areas The blue and red arrows represent the surprise and uncertainty values respectively in each 8 types of chord progressions. The blue-to-red gradients represent the number of clicks. The blue and red arrows represent the surprise and uncertainty values, respectively. Four of the 8 types began with three chords, each with low surprise and uncertainty (A–D), while the other four began with three chords each displaying high surprise and uncertainty (E–H). For each chord progression, the last (i.e., 4th) chord was generated with a 2 × 2 pattern, varying in uncertainty and surprise. The first pattern exhibited both low surprise and low uncertainty (A and E), the second had low uncertainty but high surprise (B and F), the third showcased low surprise with high uncertainty (C and G), and the fourth possessed both high surprise and uncertainty (D and H).

We examined how the total number of clicks at cardiac and abdomen positions were correlated with the scores of valences, arousals, and aesthetic appreciation. The Shapiro–Wilk test for normality showed the violations of the assumption of normality on all the data (p < 0.001). Hence, we applied the Spearman correlation tests. All the results of statistical analyses and the descriptive have been deposited to an external source. All the figures are shown in Figure S5 of the supplemental information. Results revealed significant positive correlations of valence with the number of clicks localized to the cardiac area only in the sLuL-sHuL sequence (Figure 4B) (rs = 0.132, p < 0.001). This suggests that cardiac sensation is an important factor for positive emotion in this type of chord progression.

We also carried out correlation analyses to explore the relationship between emotions (valence, arousal, 33 categorical emotions) and bodily sensation by using the data combining clicks across chord progression. Comprehensive results and descriptive statistics are available at an external repository (https://osf.io/cyqhd/). Notably, our findings revealed a significant correlation between the intensity of sensations in the heart and positive valence (rs = 0.071, p < 0.001). In contrast, sensations in the abdomen and head did not demonstrate a significant correlation with valence. This suggests a strong association between cardiac sensations and feelings of pleasure. Interestingly, pronounced sensations in the head were significantly associated with specific negative emotions, namely anxiety (rs = 0.066, p < 0.001) and confusion (rs = 0.056, p < 0.001).

We also employed mediation analysis to understand whether and how the influence of listening to different chords on bodily sensations is mediated by emotional experiences, and vice versa. The analysis was conducted iteratively for different combinations of independent variables (chords), mediators (either valence or bodily sensation), and dependent variables (either valence or bodily sensation). Comprehensive results and descriptive statistics are available at an external repository (https://osf.io/cyqhd/). For the sLuL-sLuL (Figure 1A) and the sLuL-sHuL sequence (Figure 1B), cardiac sensations were significantly and positively mediated by the emotion of valence. Specifically, in the sLuL-sLuL sequence, the indirect effect of valence on cardiac sensations was quantified as 0.0294 (95% CI: 0.0140 to 0.0463). For the sLuL-sHuL sequence, the indirect effect was determined to be 0.0176 (95% CI: 0.0083 to 0.0292). However, while valence mediates cardiac sensations, these sensations do not subsequently mediate the valence.

For the sHuH-sHuH (Figure 1H), sHuH-sHuL (Figure 1F), and sHuH-sLuL (Figure 1E) sequences, cardiac sensations were significantly mediated in a negative direction by the emotion of valence. To specify, the indirect effect for the sHuH-sHuH sequence was −0.0125 (95% CI: −0.0229 to −0.0047). For the sHuH-sHuL sequence, this effect was −0.0153 (95% CI: −0.0262 to −0.0067), and for the sHuH-sLuL sequence, it was −0.0101 (95% CI: −0.019 to −0.0035).

In summary, our results indicate that the sLuL-sLuL (Figure 1A) and sLuL-sHuL (Figure 1B) sequences, when inducing positive valence, elicit cardiac sensations. Conversely, the sHuH-sHuH (Figure 1H), sHuH-sHuL (Figure 1F), and sHuH-sLuL (Figure 1E) sequences, upon evoking positive valence, result in a decrease in cardiac sensations.

### Emotional distribution in response to each musical chord progression

The emotional distributions of 8 chord progressions were shown in Figure S4 in the supplemental information. Given prior research by Cowen and colleagues,^42^^,^^43^^,^^44^ it is understood that the higher-order emotions elicited by auditory and visual stimuli can be characterized by a distribution of distinct emotions. Based on this premise, we sought to determine how each musical chord stimulus could be characterized in terms of these emotional distributions. Our primary aim was to identify any unique emotional distributions associated with pronounced sensations in the cardiac and abdominal regions, believing that this would provide a deeper understanding of the constituent emotions closely tied to interoceptive perception. To visualize the difference in the emotional distributions between the different types of chord progressions, between groups who rated high and low valence, and between groups who showed strong and weak sensations of each cardiac and abdomen area, we compared the scores of the emotional distributions of different emotional categories by Uniform Manifold Approximation and Projection (UMAP).^45^^,^^46^

**Figure 5:**
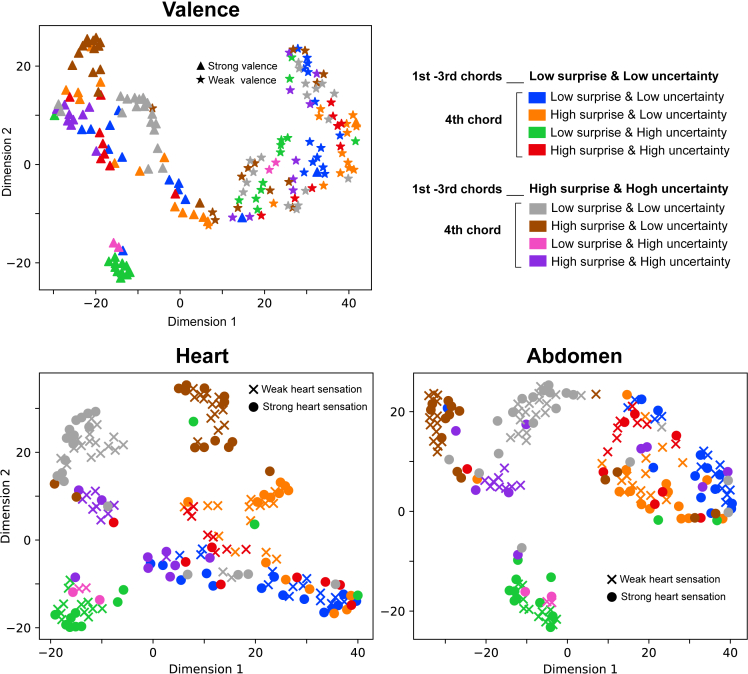
The structure of reported emotional experience A chromatic map of average emotional responses to 92 chord progressions within a 33-dimensional categorical space of reported emotional experience. Uniform Manifold Approximation and Projection (UMAP) was applied to the loadings of the 92 chord progressions on the 33 categorical judgment dimensions, generating loadings of each stimulus on two axes.

The different emotional categories were separately averaged for each of the 92 distinct chord progressions included within the eight types, as well as for groups with valence scores above 6 and below 4 (Figure 5, top). Additionally, the emotional categories were separately averaged for those who experienced bodily sensations with a click count greater than 1 in the cardiac area and for those who did not experience bodily sensations with a click count of 0 in the cardiac area (Figure 5, bottom left). Lastly, the emotional categories were separately averaged for those who experienced bodily sensations with a click count greater than 1 in the abdominal area and for those who did not experience bodily sensations with a click count of 0 in the abdominal area (Figure 5, bottom right).

Then, using the averaged data from these emotional categories, we employed UMAP in Python to craft a two-dimensional representation. The results showed that the intricate interplay between musical uncertainty, prediction error, and temporal dynamics underlies distinct emotional components. Notably, even when different types of chord progressions culminate in a singular positive valence, the dynamics of musical chords, especially the sLuL-sLuH sequence, manifest uniquely.

## Discussion

The present study examined how perceptions of musical chord progressions elicit bodily sensations and emotions. Specifically, within the framework of predictive processing, we aim to determine how chord prediction and its uncertainty evoke interoceptive bodily sensations, particularly around the cardiac and abdominal regions. Our findings indicate that the sLuL-sHuL sequence (Figure 2B) representing the condition where the 1st-3rd chords have low surprise and uncertainty and the 4th chord has high surprise and low uncertainty and sLuL-sLuL sequence (Figure 2A) representing the condition where the 1st-3rd chords have low surprise and uncertainty and the 4th chord has low surprise and uncertainty provoked cardiac and abdomen bodily sensations, respectively. Further, GLMM analysis demonstrated that sensations in the cardiac and abdominal regions are independent, suggesting distinct underlying mechanisms for these two sensations. Such bodily sensations were correlated with an aesthetic appreciation (Figure 3). This effect was pronounced when the preceding musical context comprised predictable and less uncertain chords (Figures 2A–2D), in contrast to sequences typified by high prediction errors and high uncertainty (Figures 2E–2H). Notably, in the sLuL-sHuL sequence (Figure 2B) representing the condition where the 1st-3rd chords have low surprise and uncertainty and the 4th chord has high surprise (Figure 4B), the intensity of cardiac sensations was positively correlated with valence. Further, the mediation analysis indicated that this sequence prompts cardiac sensations through the mediation of positive valence.

Building on the foundation of previous research, our study provides insights into the relationship between musical perception, interoception, and the resulting emotional experiences. The distinct bodily sensations observed in response to different chord progressions underscore the importance of understanding the nuances of musical uncertainty and surprise in the context of predictive processing. The observed patterns of bodily sensations, especially in the cardiac and abdominal regions, suggest a deep-rooted connection between musical perception and interoceptive awareness. This connection is further emphasized by the correlation between these sensations and positive valences and mediation of valence for connecting between musical chord sequence and cardiac sensation. Particularly in the sLuL-sHuL sequence, the intensity of cardiac sensations was positively correlated with valence. This may suggest that the pronounced cardiac sensation may be a key factor in eliciting musical enjoyment specifically in a specific type of chord progression.

Notably, we found a significant correlation between the intensity of sensations in the heart and positive valence. Further, the sLuL-sLuL and sLuL-sHuL sequences, but not other sequences, enhance cardiac sensations through the mediation of positive valence. Conversely, sensations in the abdomen and head did not exhibit such a relationship with valence. This finding implies a robust connection between cardiac sensations and pleasurable feelings. Intriguingly, heightened sensations in the head were significantly correlated with particular negative emotions, such as anxiety and confusion. Additionally, our data indicated that the sLuL-sLuL and sLuL-sHuL sequences predominantly evoked interoceptive sensations in the abdomen and heart. However, these sequences induced more subdued sensations in the head compared to other sequences. Strong cardiac sensations might be linked to positive valence and diminished sensations in the head, while intensified sensations in the head could be associated with specific negative emotions, such as anxiety. Nevertheless, due to the emergence of correlations with multiple emotions and the extensive sample size, which yielded weak correlation coefficients, a more detailed examination of the interplay between bodily sensations and emotions appears to be essential.

Our past study investigating the bodily mapof sound pitch revealed that the lowest bodily location in response to pitch was not the feet, located at the lowest point of the body, but rather the abdomen, the lowest part of the visceral system.^47^ Thus, emotional experience induced by auditory perception has been suggested to involve the 'embodiment' of sound through proprioceptive and interoceptive pathways. In this study, by specifically employing musical stimuli of chords instead of simple pitches, we more definitively highlighted interoceptive bodily sensations including the cardiac (heart) and abdominal (stomach) regions.

The interplay between musical uncertainty, prediction error, and temporal dynamics is central to our understanding of how music elicits specific emotional and bodily responses. Our findings suggest that the temporal dynamics of musical chords, especially those transitioning from predictable chords to those characterized by high uncertainty and low surprise, play a pivotal role in shaping our emotional experiences. This understanding of musical emotions as a temporal art form offers a fresh perspective on the age-old question of why music moves us in the ways that it does.

A past study detected that chords combining low uncertainty with high surprise or those combining high uncertainty with low surprise were the most pleasurable.^14^ This suggests that the musical pleasure forms a two-dimensional inverted U curve based on uncertainty and surprise. However, music entails extended contexts that cannot be fully captured by the simple prediction and uncertainty between one chord and the next. This context can influence the perception of even the same two-chord sequence, resulting in different emotional experiences depending on the surrounding context. For example, if we only looked at first-order transitional probability and uncertainty, the sLuL-sLuL sequence (Figure 1A) and sHuH-sLuL sequence (Figure 1E) were considered the same type of chord. However, as evident from the bodily mapresults in this study (Figure 2), the body maps for the sLuL-sLuL sequence and sHuH-sLuL sequence are distinctly different. Notably, while the sLuL-sLuL sequence shows bodily sensations in the abdominal region, such sensations are absent in the sHuH-sLuL sequence. Furthermore, regarding results on emotions (Figure 3), the sLuL-sLuL sequence significantly exhibits higher positive valence and aesthetic appreciation compared to the sHuH-sLuL sequence. These findings suggest that contextual temporal dynamics can lead to variations in interoceptive sensations and emotions.

This study found that the different types of chord progressions elicited the distinct feeling of categorical emotions. Notably, while the sLuL-sLuL and sLuL-sHuL sequences evoked aesthetic appreciation, they lead to decreasing of the negative emotions of awkwardness and anxiety (Figure S4 in the supplemental information). Further, the predictable chord progressions of sLuL-sLuL elicited feelings of calmness, relief, satisfaction, nostalgia, and empathy.

Consequently, in addition to the two dimensions of uncertainty and surprise, it is necessary to elucidate musical prediction and emotion through a three-dimensional model that takes into account the preceding contexts (more dynamics). The present study revealed that the emotional responses induced by uncertainty-weighted prediction error could be modulated by preceding musical contexts.^48^ In other words, the temporal dynamics of uncertainty-weighted prediction error could offer a more nuanced understanding of musical emotions as a temporal art form.

We also found that the three-dimensional interplay between musical uncertainty, prediction error, and temporal dynamics underlies distinct emotional components. Interestingly, even when different types of chord progressions culminate in a singular positive valence, the different temporal dynamics (fluctuation) of surprise and uncertainty manifest uniquely. It’s worth acknowledging the diverse forms of musical pleasure and distinguishing between these could elucidate distinct effects on our mind and body.

Our findings also suggest that the different interplay of uncertainty and prediction error elicits distinct emotional distributions, especially in the sLuL-sLuH sequence representing the condition where the 1st-3rd chords have low surprise and uncertainty and the 4th chord has low surprise and high uncertainty (Figure 5), even when all these chord progressions converge on a positive valence. Further, a certain emotional profile may lead to sensations in the cardiac area, particularly pronounced in the sHuH-sLuL sequence (distinguished by the gray circle compared to the gray cross in Figure 5). This sequence is characterized by high surprise and uncertainty in its initial three chords, transitioning to a fourth chord marked by low surprise and uncertainty. As illustrated in Figure 2E, the sHuH-sLuL sequence predominantly evokes sensations localized to the cardiac region. This chord progression conveys a palpable tension (or prediction error) through its first three chords, which is abruptly alleviated by the predictability of the fourth chord. Such marked shifts in prediction could potentially evoke pronounced interoceptive sensations.

This study proposes a hypothesis for emotion generation from auditory perception, emphasizing the embodiment of sound through predictive processing. That is, we postulate that positive emotions emerge during the process of sound embodiment, wherein auditory bodily sensations become localized, i.e., prediction errors are minimized, through proprioceptive and interoceptive predictive processing. Through computational modeling, a past study indicated that individuals exhibiting hypo-sensitivity to stimuli, as seen in depression or alexithymia,^49^^,^^50^^,^^51^ might struggle to adapt to auditory sensory signals.^37^ Consequently, the internal model’s updating mechanism, aimed at minimizing prediction errors, may become dysfunctional. Given these findings, we observed the less localized body maps and amplified anxiety in prior research on alexithymia and depression^47^^,^^52^^,^^53^ may be attributed to a malfunctioning error-minimization mechanism due to hypo-sensitivity. This malfunction might subsequently lead to emotions not being correctly identified, giving rise to anxiety.

The sLuL-sLuL and sLuL-sHuL sequences primarily elicited interoceptive sensations in the abdomen and heart. Particularly, these sequence prompts cardiac sensations through the mediation of positive valence. In contrast, these sequences induced milder sensations in the head, including the ears, compared to other sequences. The head region might be associated with exteroception, especially hearing. Intense interoceptive sensations might lead to diminished exteroceptive bodily sensations. Future studies should aim to further elucidate the fundamental distinctions between exteroceptive and interoceptive body maps. Beyond the exteroception such as hearing, music induces feelings inside the body such as interoception and proprioception. Particularly, interoceptive awareness has been identified as being integral to our mental well-being.^7^ Thus, profound interoceptive changes induced by music might potentiate interoceptive awareness, thereby potentially benefiting mental health.

This study demonstrated that emotional responses to music based on predictive processing can induce specific bodily sensations. Particularly, even when different types of chord progressions culminate in a single positive valence, the varying temporal dynamics of surprise and uncertainty manifest uniquely. It is important to acknowledge the diverse forms of musical pleasure, and distinguishing between these could shed light on their distinct effects on our minds and body.

### Limitations of the study

It is worth noting that there is variation in the number of chord progressions within each of the eight groups. To address the potential bias stemming from this variability, we balanced the dataset by considering the ratio of participants’ responses within each group. This balancing approach helps address the issue of unequal data distribution and enhances the statistical robustness of our analysis. However, it is important to acknowledge that we cannot entirely eliminate the possibility of bias affecting the results. This study did not directly investigate whether interoceptive awareness was enhanced by the perception of musical chords. In other words, it is possible that bodily sensations were simply induced in the chest and abdominal areas regardless of interoception. That is, our experimental paradigm of having participants click on a bodily mapmay not adequately capture the nuances of interoceptive experiences. While this approach provides a visual representation of perceived sensations, it may not directly reflect the depth or quality of interoceptive awareness. The choice of this task was driven by our aim to provide an intuitive and accessible method for participants to report their sensations. While it might not offer the granularity of more specialized interoceptive tasks, it serves as a bridge between subjective experiences and more objective measures. Future research should investigate interoception such as a heartbeat detection test before and after music listening to determine whether interoceptive awareness is enhanced.

### Conclusions

This study reveals the intricate interplay between musical uncertainty, prediction error, and temporal dynamics in eliciting distinct bodily sensations and emotions. We found that specific types of chord progressions provoke interoceptive sensations in the cardiac and abdominal regions. These sensations are associated with aesthetic appreciation. Furthermore, our findings underscore the importance of acknowledging diverse forms of musical pleasure, each with unique effects on our minds and bodies. We propose a hypothesis for emotion generation through predictive processing and sound embodiment, suggesting a potential link between musical interoception and mental well-being. Further research is required to explore this connection and to differentiate between exteroceptive and interoceptive bodily sensations in music perception.

## STAR★Methods

### Key resources table

**Table:** 

REAGENT or RESOURCE	SOURCE	IDENTIFIER
**Software and algorithms**		

Matlab 2022b	https://jp.mathworks.com/products/matlab.html	RRID:SCR_001622
GORILLA Experiment Builder	https://gorilla.sc/	RRID:SCR_020991
Python 3.1.1	www.python.org	RRID:SCR_008394

### Resource availability

#### Lead contact

Further information and requests for resources should be directed to and will be fulfilled by the Lead Contact, Tatsuya DAIKOKU (daikoku.tatsuya@mail.u-tokyo.ac.jp).

#### Materials availability

This study did not generate new unique reagents.

#### Data and code availability

• All of the anonymized raw data files, stimuli used in this study, and the results of statistical analysis have been deposited to an external source (https://osf.io/cyqhd/) and are publicly available as of the date of publication. The other data are shown in supplementary data.
• This paper does not report original code.
• Any additional information required to reanalyze the data reported in this paper is available from the lead contact upon request.

### Experimental model and study participant details

The experiment was conducted in accordance with the guidelines of the Declaration of Helsinki and was approved by the Ethics Committee of The University of Tokyo (Screening number: 21-335). All participants gave their informed consent and conducted the experiments by on a PC.

The present study consists of body-mapping tests and the following emotional judgements for every one of the eight types of 4-chord progression. The participants consisted of people who had no history of neurological or audiological disorders and no absolute pitch (N = 527, *M*_age_±SD = 32.18±5.37, female = 257, specific musical training±SD = 2.47±5.66).

### Method details

The experimental paradigm was generated using Gorilla Experiment Builder (https://gorilla.sc), which is a cloud-based research platform that allows the deploying of behavioural experiments online. Each participant was provided with eight types of chord progressions including 4 chords (500ms/chord, 44.1kHz, 32bit, Electric Piano 1 based on the General MIDI, amplitude based on equal-loudness-level contours).

We used a statistical-learning model^37^^,^^38^ to derive the surprise and uncertainty of every chord. This model computes the Shannon information content and entropy based on transitional probabilities^39^ of each chord from a corpus of 890 pop songs in the McGill Billboard Corpus.^40^ The statistical learning is an implicit process by which the brain automatically computes transitional probability from sequences, grasps uncertainty/entropy, and predicts a future state based on the internalized statistical model.^54^^,^^55^ The transitional probability is a conditional probability of an event en+1, given the preceding n events based on Bayes' theorem (P(e_n+1_|e_n_)). From a psychological perspective, the transitional probability (P(e_n+1_|e_n_)) can be interpreted as positing that the brain predicts a subsequent event e_n+1_ based on the preceding events e_n_ in a sequence. In other words, learners expect the event with the highest transitional probability based on the latest n states, whereas they are likely to be surprised by an event with a lower transitional probability. Furthermore, transitional probabilities are often translated as information contents:

$$I\left(e_{n+1}\right)=–\log_{2}P\left(e_{n}+1|e_{n}\right)$$

The lower information content (i.e., higher transitional probability) means higher predictabilities and smaller surprising, whereas the higher information content (i.e., lower transitional probability) means lower predictabilities and larger surprising. In the end, a tone with lower information content may be one that a composer is more likely to predict and choose as the next event, compared to tones with higher information content. The information content can be used in computational studies of music to discuss psychological phenomena involved in prediction and statistical learning. The entropy of chord e_n+1_ is the expected information content of chord e_n_. This is obtained by multiplying the conditional probability of all possible chords in S by their information contents and then summing them together, giving:

$$H\left(e_{n+1}\right)=-\sum_{p}\left(e_{n+1}=e|e_{n}\right)\log_{2}p\left(e_{n+1}=e|e_{n}\right)$$

Entropy gauges the perceptual uncertainty a listener feels in predicting an upcoming chord based on prior chords, while information content quantifies the surprise experienced upon hearing the actual chord. Using this model, we generated the 92 unique chord progressions encompassed within the eight types of chord progressions. All the chord progressions have been deposited to an external source (https://osf.io/cyqhd/). Each type is characterized by varying degrees of uncertainty and surprise (see red and blue lines in Figure 2). They consist of 1) sLuL-sLuL sequence (Figure 2A) representing the condition where the 1st-3rd chords have low surprise and uncertainty and the 4th chord has low surprise and uncertainty, 2) sLuL-sHuL sequence (Figure 2B) representing the condition where the 1st-3rd chords have low surprise and uncertainty and the 4th chord has high surprise and low uncertainty, 3) sLuL-sLuH sequence (Figure 2C) representing the condition where the 1st-3rd chords have low surprise and uncertainty and the 4th chord has low surprise and high uncertainty, 4) sLuL-sHuH sequence (Figure 2D) representing the condition where the 1st-3rd chords have low surprise and uncertainty and the 4th chord has high surprise and uncertainty, 5) sHuH-sLuL sequence (Figure 2E) representing the condition where the 1st-3rd chords have high surprise and uncertainty and the 4th chord has low surprise and uncertainty, 6) sHuH-sHuL sequence (Figure 2F) representing the condition where the 1st-3rd chords have high surprise and uncertainty and the 4th chord has high surprise and low uncertainty, 7) sHuH-sLuH sequence (Figure 2G) representing the condition where the 1st-3rd chords have high surprise and uncertainty and the 4th chord has low surprise and high uncertainty, and 8) sHuH-sHuH sequence (Figure 2H) representing the condition where the 1st-3rd chords have high surprise and uncertainty and the 4th chord has high surprise and uncertainty.

In other words, the four of the 8 types began with three chords, each with low uncertainty and surprise (Figures 2A–2D), while the other four began with three chords each displaying high uncertainty and surprise (Figures 2E–2H). For each set of these four progressions, the fourth chord was generated with a 2x2 pattern, varying in uncertainty and surprise. Specifically, there are four variations for the fourth chord: the first exhibited both low uncertainty and surprise (Figures 2A and 1E), the second had low uncertainty but high surprise (Figures 2B and 1F), the third showcased high uncertainty with low surprise (Figures 2C and 1G), and the fourth possessed both high uncertainty and surprise (Figures 2D and 1H). The thresholds for the high and low values were established based on the top and bottom 20% of all data points for both uncertainty and surprise. Multiple chord progressions were generated for each of the eight types (sLuL-sLuL: 14, sLuL-sHuL: 14, sLuL-sLuH:12, sLuL-sHuH: 8, sHuH-sLuL: 18, sHuH-sHuL: 14, sHuH-sLuH: 3, sHuH-sHuH: 9), and the chord progression employed was randomly selected for each participant.

Participants were exposed to these eight types of chord progressions in random order. Following each listening session, they were asked to respond within 10 seconds with clicks to the position in the body where they felt sensations from the chords, using the body image presented on the screen. The clicking was allowed any number of times up to a maximum of 100 clicks, and clicking while listening to the sound was also allowed (see Figure S1 in the supplemental information for the details). Two surveys were used to obtain emotional judgements. The first comprised multiple-choice categorical judgements; that is, in each type of chord progression, participants were required to select the best 5 emotional categories in ranking elicited by each sound from a list of 33 categories (see Table S1 in the supplemental information). The 33 emotion categories were derived from emotion taxonomies of prominent theorists, Keltner and Lerner^56^ and based on the previous study by Cowen et al.^42^^,^^43^^,^^44^ The second kind comprised nine-point dimensional judgments; that is, after hearing the chord progression, participants were required to rate each type of chord progression along the valence and arousal. Each rating was obtained on a nine-point Likert scale with the number 5 anchored at neutral.

### Quantification and statistical analysis

Using the coordinate data of x and y in the body mapping test, we extracted the total number of clicks at two interoceptive positions including cardiac and abdomen areas in each participant. The raw data of x and y coordinates (see Figure S2 in the supplemental information) were down-sampled by a factor of 40. The body image template used had a pixel size of 871 pixels in width and 1920 pixels in height. Specific regions were demarcated to represent distinct bodily areas: the cardiac region spanned a width from 360 to 550 pixels and a height from 390 to 620 pixels, while the abdominal region was delineated between 360 to 510 pixels in width and 650 to 850 pixels in height. The Figures of the body topographies (Figure 2) were generated using Matlab (2022b) by interpolating the coordinates of x and y in a meshgrid format with a colour map that represented the neighbouring points. As depicted in Figure 2, it was observed that the clicks were predominantly concentrated in the heart and abdominal regions. The results of the best 5 emotional categories in the ranking were used to score the intensity of 33 emotions. That is, the first, second, third, fourth, and fifth categories were each scored as a 5, 4, 3, 2, and 1 point respectively. The scores of each 33 emotional categories were then averaged for all participants (see Figure S4 in the supplemental information for all results).

The click data may exhibit dependencies between sensations in one area and those in others both in terms of spatial autocorrelation, where individuals may indicate sensations over large areas, and in terms of individual differences, where individuals vary in the overall intensity or amount of sensation they report. To assess these individual differences and dependencies, we conducted a generalized linear mixed model analysis (GLMM). Specifically, we investigated the relationship between total clicks and those associated with the cardiac, abdominal, and head regions, as well as the relationship between cardiac and abdominal clicks. The example of model formula is as follows:

Abdomen∼Heart+(1|Subjects)

Here, "abdomen" represents the response variable, "heart" serves as the explanatory variable, and "subjects" indicate random effects. In this model, "abdomen" depends on "heart," and the correlation structure of the data within the same subjects is also taken into account. The random effects (1∣subjects)(1∣subjects) signify that there are different random effects for each subject, accounting for individual heterogeneity. This allows the model to reflect the correlation structure among data points within the same individual. Then, we also included the head positions in analysis because the bodily mapapparently showed strong sensation in this area.

Then, we performed the Shapiro–Wilk test for normality on the total number of clicks at cardiac, abdomen, and head positions in each participant, the 33 emotional scores of the multiple-choice categorical judgements, and the valence and arousal scores of the nine-point dimensional judgments. Depending on the result of the test for normality, either the parametric or non-parametric (Friedman) repeated-measure analyses of variance (Friedman’s ANOVA) were applied to compare the total number of clicks at cardiac, abdomen, and head positions, and the scores of valence, arousal, and categorical emotional scores, among different types of chord progressions. The dependent variable was the total number of clicks for each cardiac, abdomen, and head area and the scores of valence, arousal, and categorical emotional scores, and the within-subject factor was the 8 types of chord progressions. We selected p < .01 as the threshold for statistical significance and used a Durbin-Conover method and a false discovery rate (FDR) for post-hoc analysis and multiple comparisons.

Further, either the parametric or non-parametric (Spearman) correlation analysis was applied to understand how the total numbers of clicks at cardiac and abdomen positions were correlated with the scores of valence, arousal, and aesthetic appreciation. Further, we conducted correlation tests between emotion (valence, arousal, 33 categorical emotions) and bodily sensation by using the data combining clicks across chord progression. Statistical analyses were conducted using jamovi Version 1.2 (The jamovi project, 2021). We selected *p* < .05 as the threshold for statistical significance and used an FDR method for multiple comparison testing.

We employed mediation analysis to discern the pathways through which one independent variable could influence a dependent variable via a mediator. This was vital in understanding whether and how the influence of listening to different chords on bodily sensations is mediated by emotional experiences, and vice versa. The mediation model adopted robust regression for the mediator against the independent variable, accounting for non-normally distributed residuals. Ordinary Least Squares (OLS) regression was used to ascertain the effects on the dependent variable. The model yielded total, direct, and indirect effects, with the indirect effect being the product of the mediator's coefficient from the robust regression and its coefficient from the OLS regression. To evaluate the significance and confidence interval of the indirect effect, bootstrap resampling with 5000 samples was conducted. This technique provided a non-parametric inference on the indirect effect. Each bootstrap sample was drawn with replacement from the original data and the indirect effect computed for each. The analysis was conducted iteratively for different combinations of independent variables (chords), mediators (either valence or bodily sensation), and dependent variables (either valence or bodily sensation).

Then, to visualize the difference in the emotional distributions between the different types of chord progressions and between groups who rated high and low valence, we compared the scores of the emotional distributions of emotional categories by Uniform Manifold Approximation and Projection (UMAP). For each of the 92 unique chord progressions encompassed within the eight types of chord progressions, the 33 emotional categories were averaged separately for groups with valence values above 6 and those below 4. In instances where the majority of emotional responses—exceeding 75%—were zeros, chosen by a very limited subset of participants, we have taken a decisive step. Given that our study aims to investigate general trends rather than individual differences, and considering the high likelihood that these values could represent random responses, we excluded emotions for which over 75% of responses were zero. This threshold of 75% was defined based on the evidence that certain emotions, like envy, were not selected in almost any chord progression, resulting in at least 75% zero scores. In the end, 10 emotion categories including aesthetic appreciation, amusement, relief, empa-thy, calmness, anxiety, awkward, confusion, satisfaction, and nostalgia were used for further analysis.. Subsequently, using the averaged data from remaining emotional categories, we employed UMAP in Python to craft a two-dimensional representation. Points in the plot were colour-coded based on the eight types of chord progressions. Furthermore, high-valence groups were represented by triangles, while low-valence groups were denoted with stars (★). Further, to visualize the difference in the emotional distributions between the different types of chord progressions and between groups who showed strong and weak sensations of each cardiac and abdomen area, we compared the scores of the emotional distributions of these categories by UMAP. For each of the 92 unique chord progressions encompassed within the eight types of chord progressions, the 33 emotional categories were averaged separately for groups with a score above 1 (i.e., felt bodily sensation. at cardiac or abdomen areas) and those with a score 0 (i.e., non-bodily sensation. at cardiac or abdomen areas). We excluded emotions for which over 75% of responses were zero. As stated above, 10 emotion categories including aesthetic appreciation, amusement, relief, empa-thy, calmness, anxiety, awkward, confusion, satisfaction, and nostalgia were used for further analysis. Subsequently, using the averaged data from the remaining emotional categories, we employed UMAP in Python to craft a two-dimensional representation. Points in the plot were colour-coded based on the eight types of chord progressions. Furthermore, high-valence groups were represented by the solid circle, while low-valence groups were denoted with a cross mark (✕). In all the UMAP analyses, the number of components was 2, the random state value was 42, the minimum distance value was 0.001, and the spread value was set to 10.

### Additional resources

This paper did not create any additional resources.

## Supplemental information

Document S1. Figures S1‒S9 and Tables S1‒S5
