# Supplementary material for: Bodily maps of uncertainty and surprise in musical chord progression and the underlying emotional response
Source: iScience. 2024 Apr 4;27(4):109498. doi: 10.1016/j.isci.2024.109498 (PMC11075058; doi:10.1016/j.isci.2024.109498)

iScience, Volume *27*

Supplemental information

Bodily maps of uncertainty and surprise in musical chord progression and the underlying emotional response

Tatsuya Daikoku, Masaki Tanaka, and Shigeto Yamawaki

Table S1. 33 categories of emotion (related to STAR Methods)

1	Admiration
2	Adoration
3	Aesthetic appreciation
4	Amusement
5	Anger
6	Anxiety
7	Awe
8	Awkwardness
9	Boredom
10	Calmness
11	Confusion
12	Contempt
13	Craving
14	Disappointment
15	Disgust
16	Empathy
17	Entrancement
18	Envy
19	Excitement
20	Fear/Horror
21	Guilt
22	Interest
23	Joy
24	Nostalgia
25	Pride
26	Relief
27	Romance
28	Sadness
29	Satisfaction
30	Sexual desire
31	Surprise
32	Sympathy
33	Triumph

Figure S1. Body image and example of the clicks to the position in the body (related to STAR Methods)

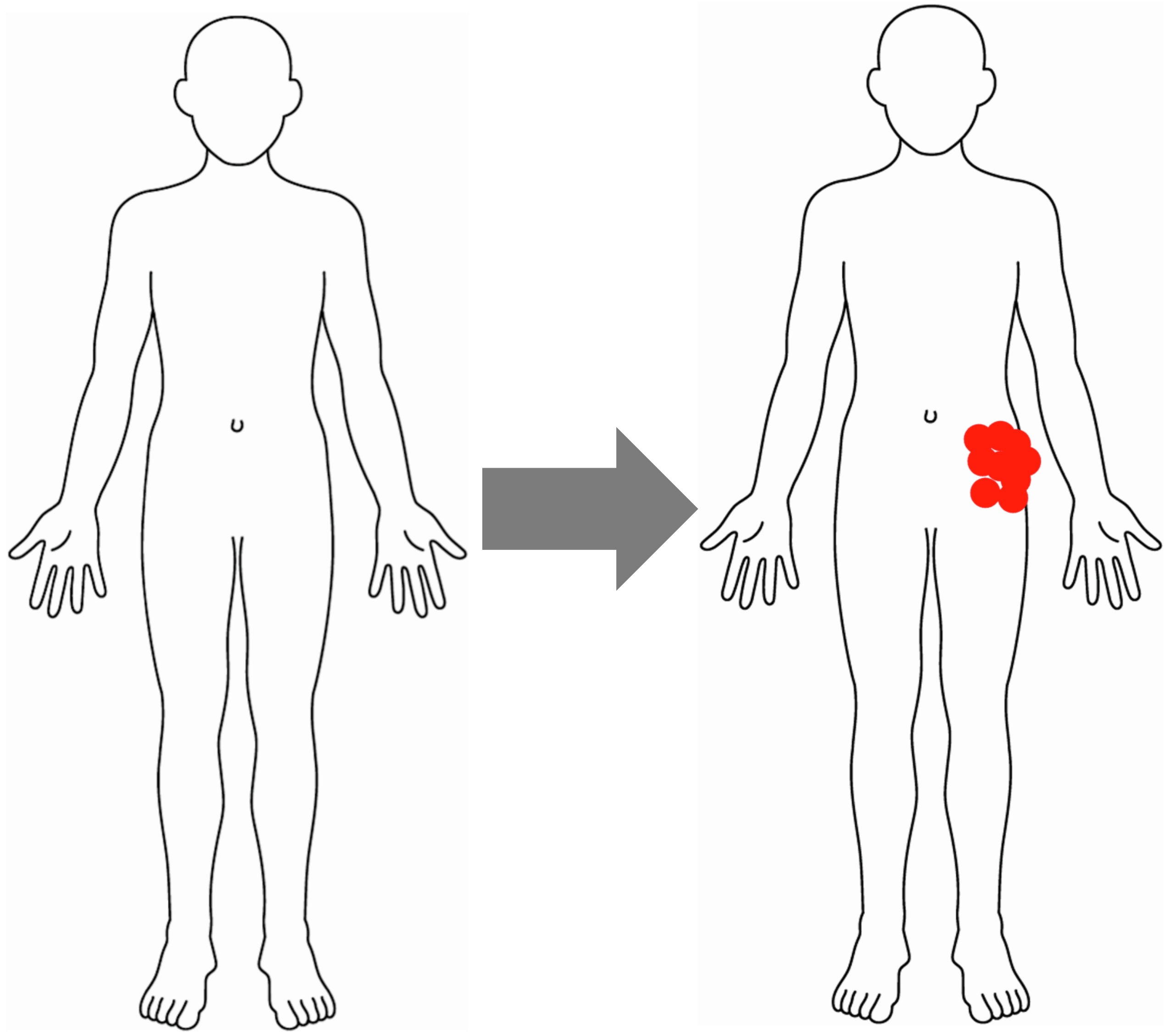

Figure S2. the row data of the click of the body (related to Figure 2 in the main text)

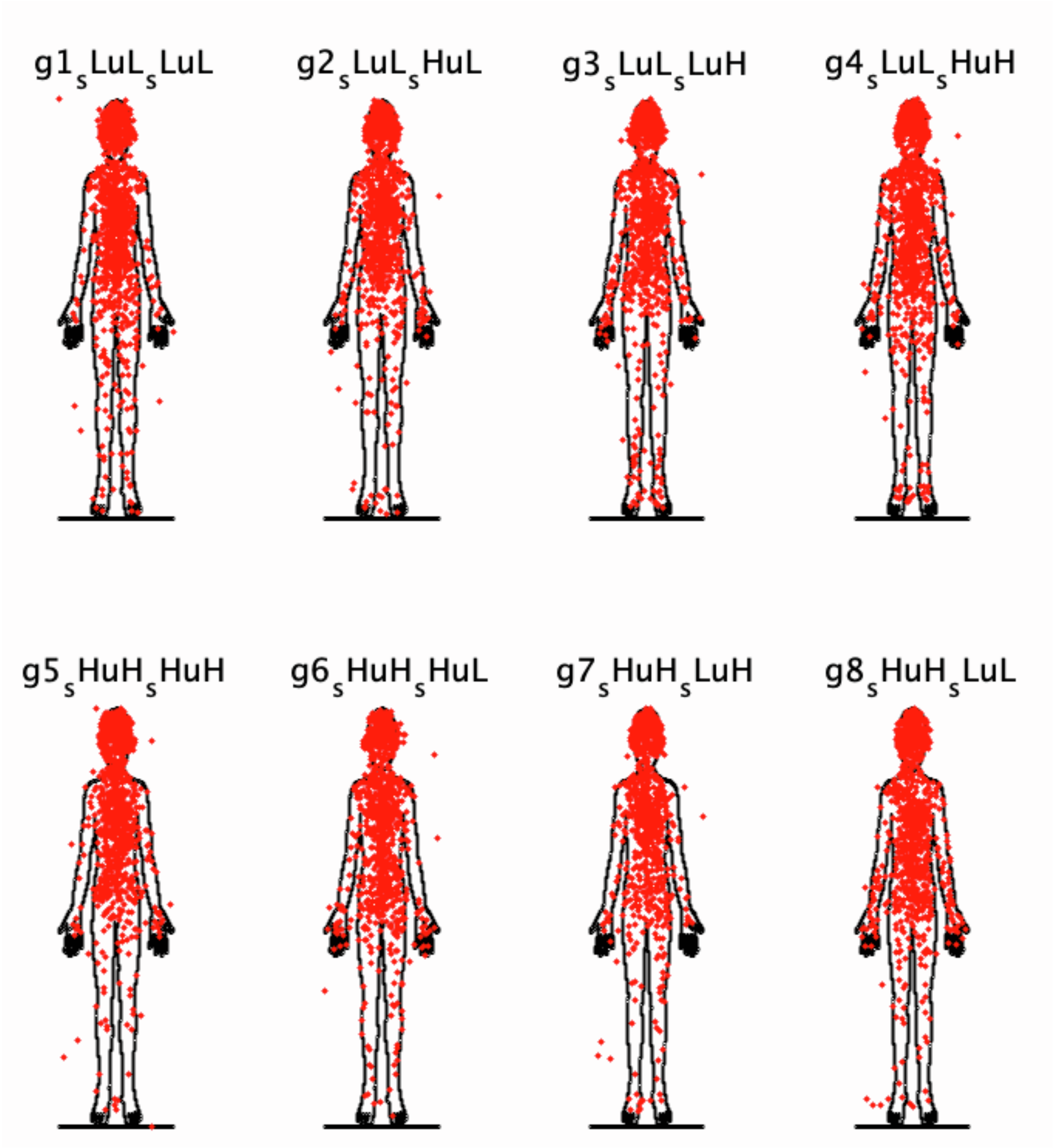

Figure S3. Valence and arousal for each type of chord progression (related to Figure 3 in the main text)

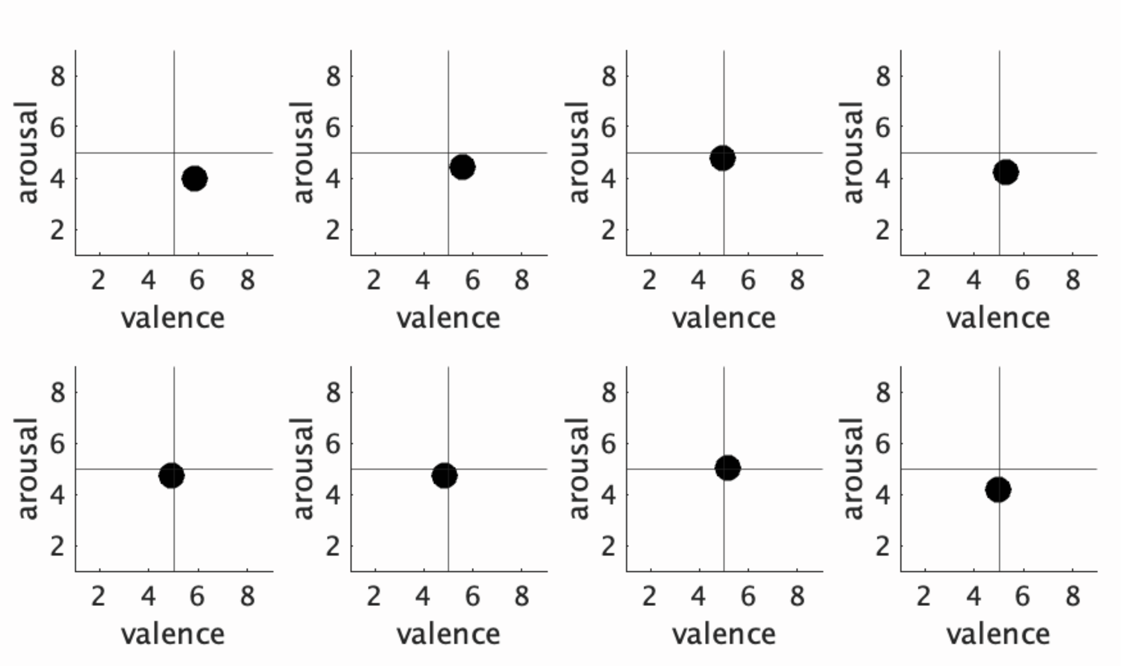

Figure S4. Categorical judgement of 33 emotions for each type of chord progression (related to Figure 3 in the main text)

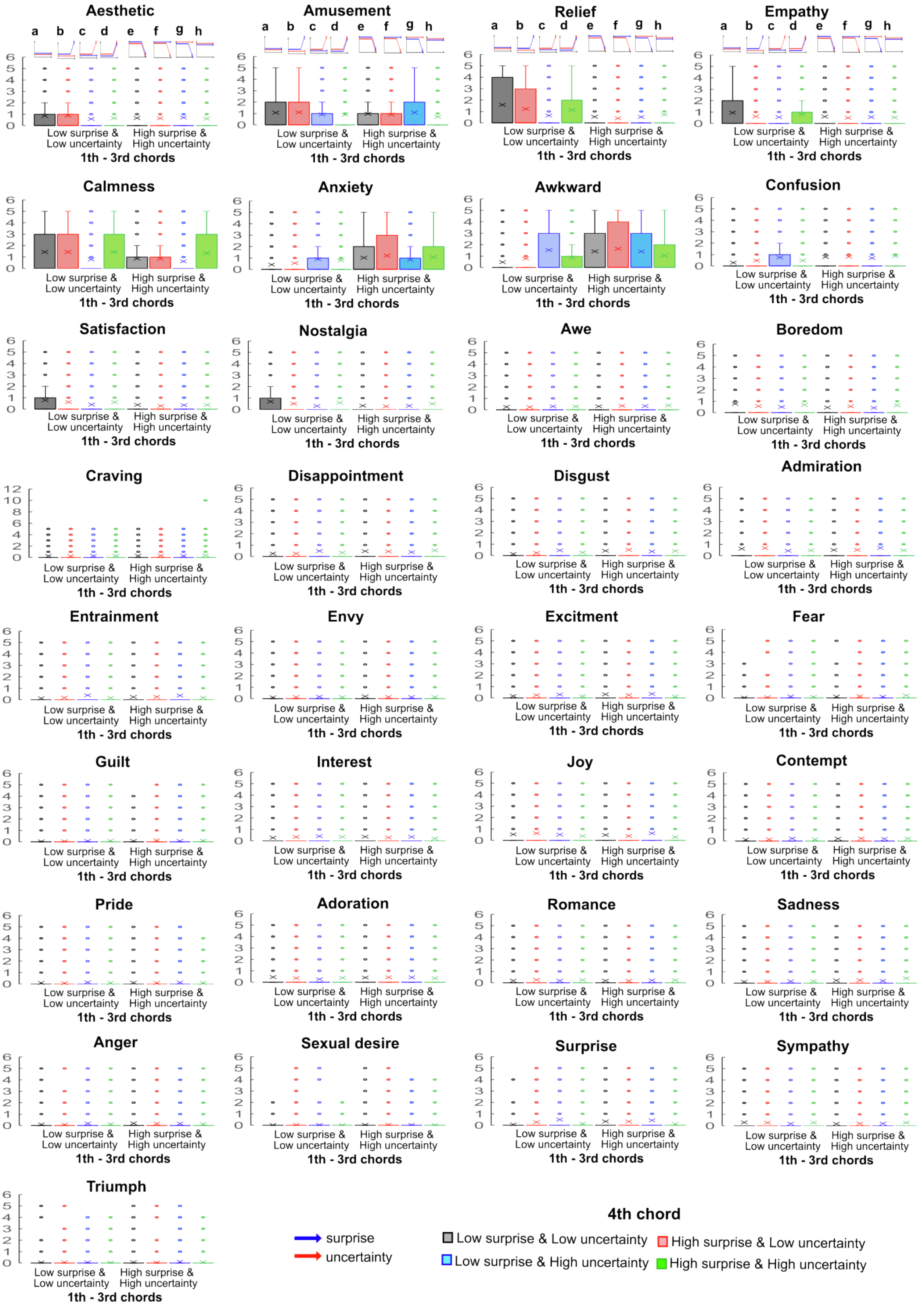

Figure S5. Correlation results for each type of chord progression (related to Figure 4 in the main text)

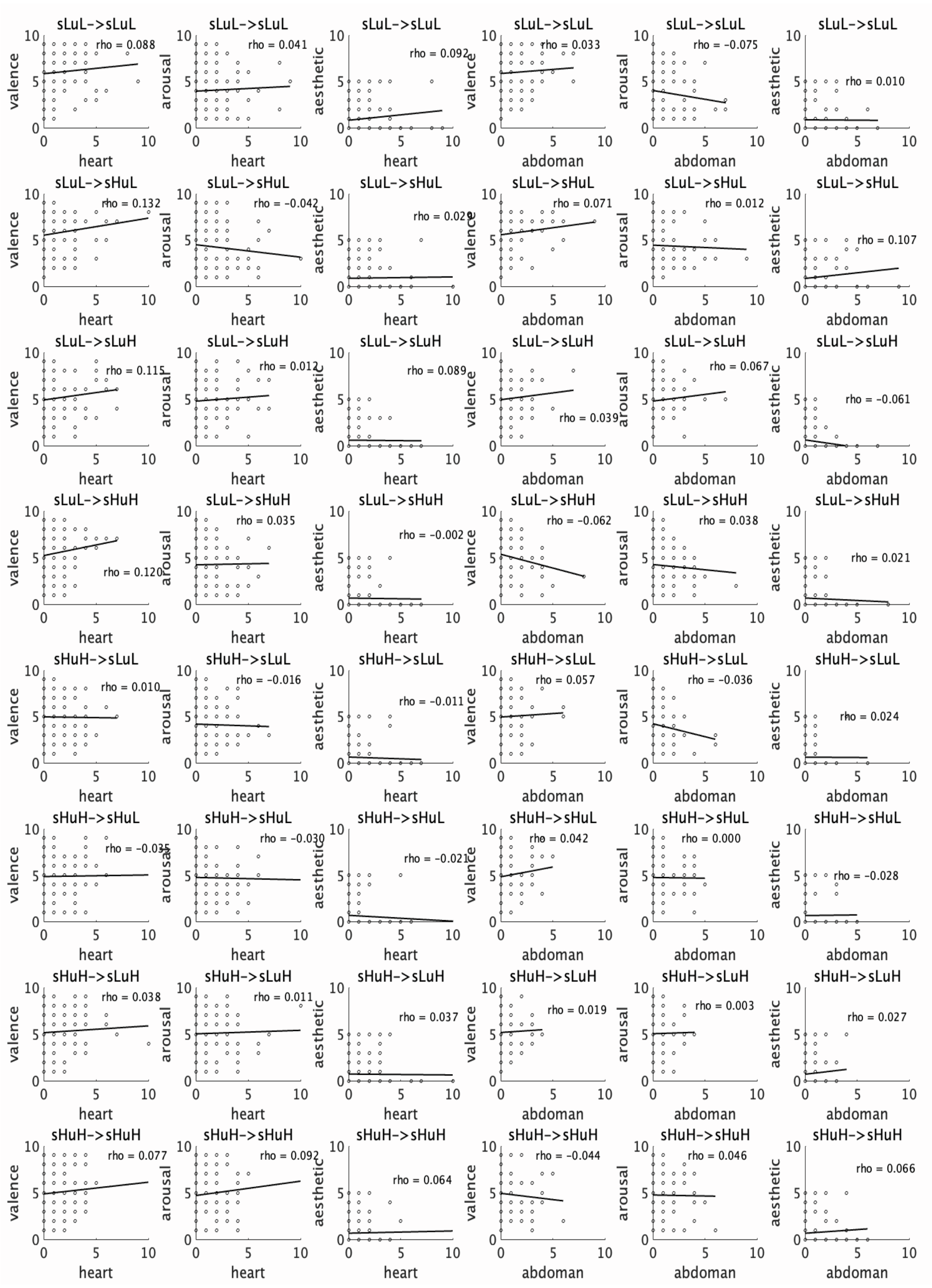

Table S2. The results of General Linear Mixed model between Heart sensation and Abdomen sensation (related to Figure 2 in the main text).

Model:	MixedLM	Dependent Variable:	Abdomen
No. Observations:	4216	Method:	REML
No. Groups:	527	Scale:	0.3699
Min. group size:	8	Log-Likelihood:	-4226.9168
Max. group size:	8	Converged:	Yes
Mean group size:	8.0		

	Coef.	Std.Err.	z	P>|z|	[0.025	0.975]
Intercept	0.200	0.018	10.927	0.000	0.164	0.235
Heart	-0.009	0.012	-0.726	0.468	-0.033	0.015
Group Var	0.119	0.019				

Figure S6. The fixed effect (a) and random effect (b) on General Linear Mixed model between Heart sensation and Abdomen sensation (related to Figure 2 in the main text).

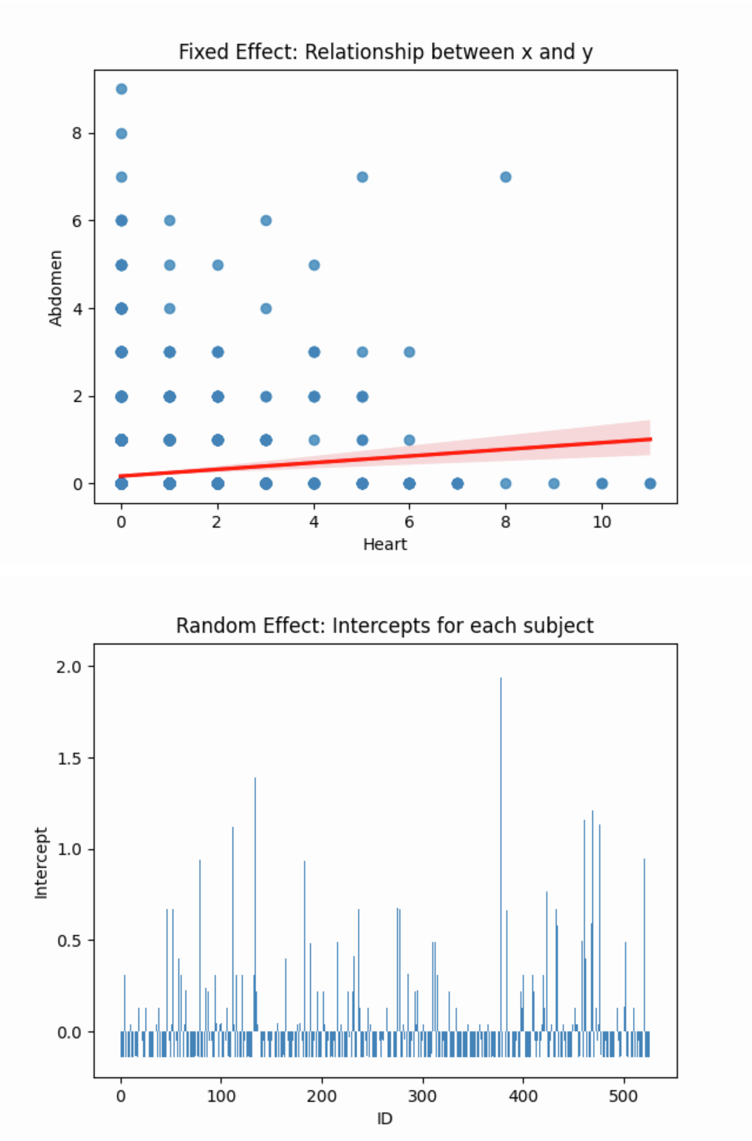

Table S3. The results of General Linear Mixed model between Total sensation and Heart sensation (related to Figure 2 in the main text).

Model:	MixedLM	Dependent Variable:	Heart			
No. Observations:	4216	Method:	REML			
No. Groups:	527	Scale:	0.5218			
Min. group size:	8	Log-Likelihood:	-5049.0808			
Max. group size:	8	Converged:	Yes			
Mean group size:	8.0					
	Coef.	Std.Err.	z	P>|z|	[0.025	0.975]
Intercept	0.040	0.028	1.409	0.159	-0.016	0.095
SUM	0.152	0.006	25.750	0.000	0.140	0.163
Group Var	0.271	0.031				

Figure S7. The fixed effect (a) and random effect (b) on General Linear Mixed model between Total sensation and Heart sensation (related to Figure 2 in the main text).

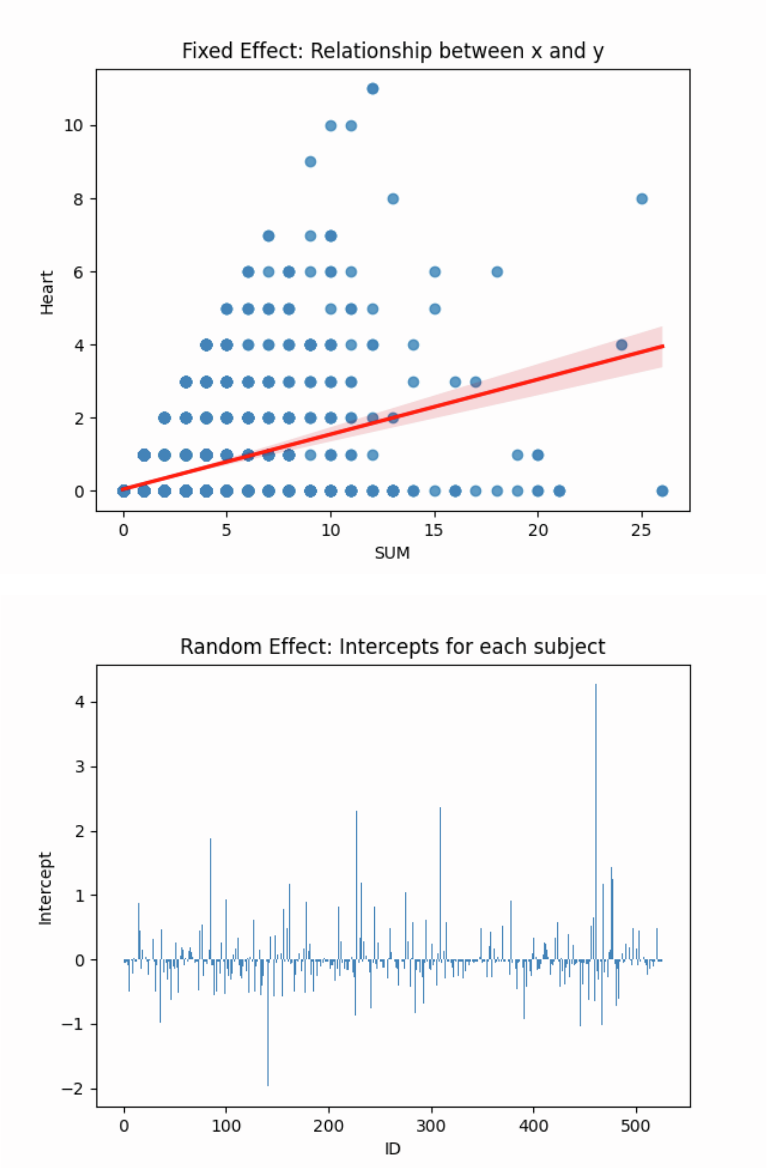

Table S4. The results of General Linear Mixed model between Total sensation and abdomen sensation (related to Figure 2 in the main text).

Model:	MixedLM	Dependent Variable:	Abdomen			
No. Observations:	4216	Method:	REML			
No. Groups:	527	Scale:	0.3483			
Min. group size:	8	Log-Likelihood:	-4057.8189			
Max. group size:	8	Converged:	Yes			
Mean group size:	8.0					
	Coef.	Std.Err.	z	P>|z|	[0.025	0.975]
Intercept	0.015	0.018	0.836	0.403	-0.021	0.052
SUM	0.083	0.004	18.963	0.000	0.074	0.092
Group Var	0.089	0.015				

Figure S8. The fixed effect (a) and random effect (b) on General Linear Mixed model between Total sensation and abdomen sensation (related to Figure 2 in the main text).

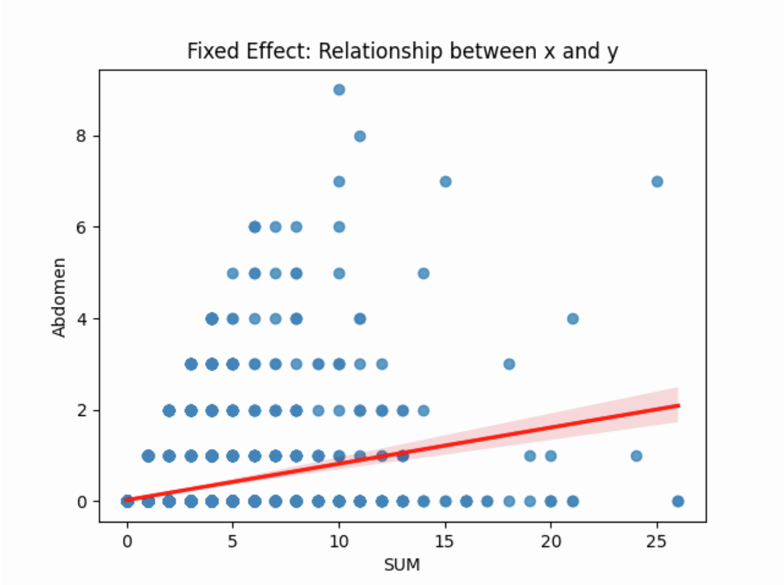

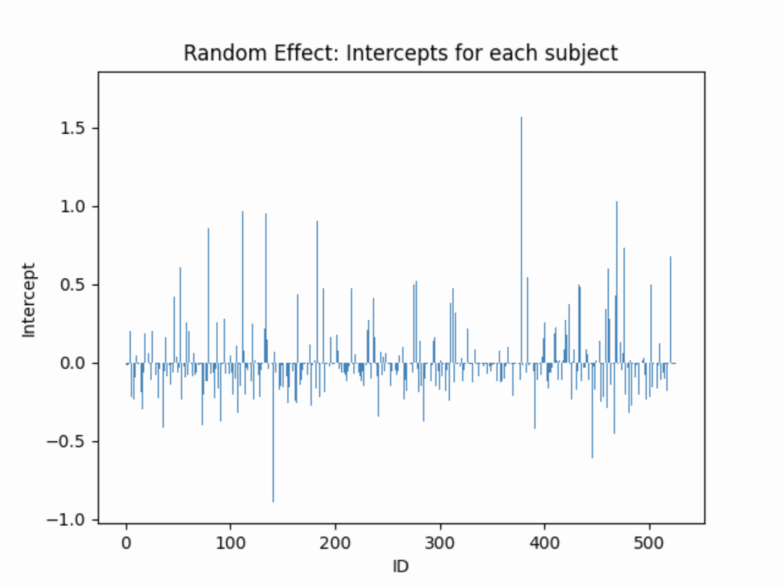

Table S5. The results of General Linear Mixed model between Total sensation and head sensation (related to Figure 2 in the main text).

Model:	MixedLM	Dependent Variable:	head			
No. Observations:	4216	Method:	REML			
No. Groups:	527	Scale:	0.7504			
Min. group size:	8	Log-Likelihood:	-6040.4246			
Max. group size:	8	Converged:	Yes			
Mean group size:	8.0					
	Coef.	Std.Err.	z	P>|z|	[0.025	0.975]
Intercept	0.174	0.050	3.509	0.000	0.077	0.271
SUM	0.298	0.008	38.124	0.000	0.283	0.313
Group Var	1.048	0.087				

Figure S9. The fixed effect (a) and random effect (b) on General Linear Mixed model between Total sensation and head sensation (related to Figure 2 in the main text).

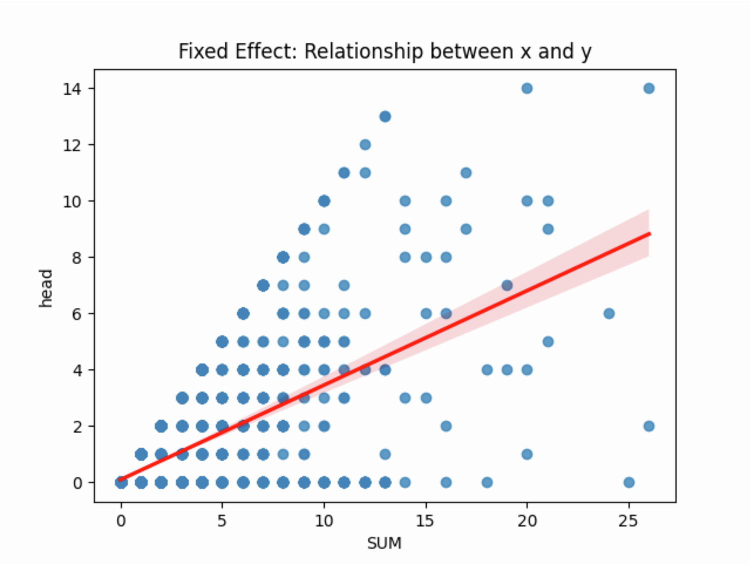

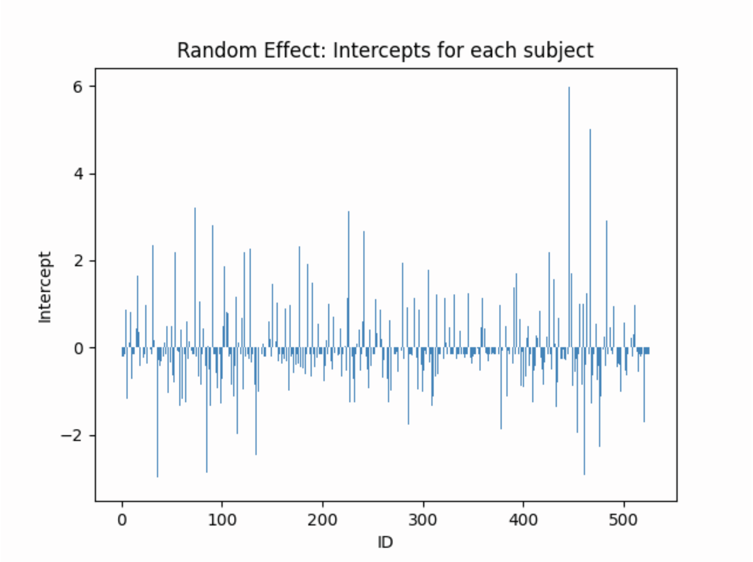

Supplement: Document S1. Figures S1‒S9 and Tables S1‒S5 [file mmc1.pdf]
